# Artificial intelligence approaches for early detection of neurocognitive disorders among older adults

**DOI:** 10.3389/fncom.2024.1307305

**Published:** 2024-02-16

**Authors:** Khalid AlHarkan, Nahid Sultana, Noura Al Mulhim, Assim M. AlAbdulKader, Noor Alsafwani, Marwah Barnawi, Khulud Alasqah, Anhar Bazuhair, Zainab Alhalwah, Dina Bokhamseen, Sumayh S. Aljameel, Sultan Alamri, Yousef Alqurashi, Kholoud Al Ghamdi

**Affiliations:** ^1^Department of Family and Community Medicine, College of Medicine, Imam Abdulrahman Bin Faisal University, Dammam, Saudi Arabia; ^2^Department of Computer Science, College of Computer Science and Information Technology, Imam Abdulrahman Bin Faisal University, Dammam, Saudi Arabia; ^3^Department of Physiology, College of Medicine, Imam Abdulrahman Bin Faisal University, Dammam, Saudi Arabia; ^4^Department of Pathology, College of Medicine, Imam Abdulrahman Bin Faisal University, Dammam, Saudi Arabia; ^5^Department of Family Medicine, College of Medicine, King Abdulaziz University, Jeddah, Saudi Arabia; ^6^Respiratory Care Department, College of Applied Medical Sciences, Imam Abdulrahman Bin Faisal University, Dammam, Saudi Arabia

**Keywords:** cognitive decline, mild cognitive impairment (MCI), dementia, artificial intelligence, logistic regression, support vector machine

## Abstract

**Introduction:**

Dementia is one of the major global health issues among the aging population, characterized clinically by a progressive decline in higher cognitive functions. This paper aims to apply various artificial intelligence (AI) approaches to detect patients with mild cognitive impairment (MCI) or dementia accurately.

**Methods:**

Quantitative research was conducted to address the objective of this study using randomly selected 343 Saudi patients. The Chi-square test was conducted to determine the association of the patient’s cognitive function with various features, including demographical and medical history. Two widely used AI algorithms, logistic regression and support vector machine (SVM), were used for detecting cognitive decline. This study also assessed patients’ cognitive function based on gender and developed the predicting models for males and females separately.

**Results:**

Fifty four percent of patients have normal cognitive function, 34% have MCI, and 12% have dementia. The prediction accuracies for all the developed models are greater than 71%, indicating good prediction capability. However, the developed SVM models performed the best, with an accuracy of 93.3% for all patients, 94.4% for males only, and 95.5% for females only. The top 10 significant predictors based on the developed SVM model are education, bedtime, taking pills for chronic pain, diabetes, stroke, gender, chronic pains, coronary artery diseases, and wake-up time.

**Conclusion:**

The results of this study emphasize the higher accuracy and reliability of the proposed methods in cognitive decline prediction that health practitioners can use for the early detection of dementia. This research can also stipulate substantial direction and supportive intuitions for scholars to enhance their understanding of crucial research, emerging trends, and new developments in future cognitive decline studies.

## 1 Introduction

Cognitive decline in the elderly population can vary widely, and the range of cognitive abilities among older individuals is influenced by various factors (Winblad et al., 2016). Normal age-related cognitive decline may include slower processing speed, occasional forgetfulness, and challenges with multitasking. These changes typically do not significantly impact daily functioning, and individuals can continue to lead independent lives. While individuals with Mild Cognitive Impairment (MCI) may experience noticeable cognitive changes, such as forgetfulness and difficulty with memory, these changes are not severe enough to interfere significantly with daily life. MCI is often considered an intermediate stage between normal age-related cognitive decline and more serious conditions such as Alzheimer’s disease (AD) or other types of dementia. Clinically, AD is characterized by a progressive decline in higher cognitive functions, including memory, thinking, language, and behavioral changes that mainly affect older people’s daily activities (Cummings-Vaughn et al., 2014).

Dementia is one of the major global health issues among the aging population. This multi-etiological syndrome is caused by different neurodegenerative diseases, including AD, which accounts for 70 to 80% of all cases of dementia (Ferencz and Gerritsen, 2015). Other types of dementia include vascular dementia, frontotemporal lobar degeneration (FTLD), and Lewy body dementia (Wang et al., 2022). The neuropathological hallmarks of AD are the abnormal accumulation of cortical extracellular senile amyloid-beta (Aβ) plaques and intracellular neurofibrillary tangles (NFTs), which selectively affect specific types of neurons and brain circuits. Also, the AD brain often displays at least moderate atrophy of the limbic lobe structures and atrophy of the gyri over the frontal and temporal lobes (Goerdten et al., 2019; Wang et al., 2021; Doblhammer et al., 2022). Several disease-modeling studies indicate that abnormal accumulation of Aβ in the brain begins 10–20 years before AD clinical symptoms (Launer et al., 1995; Kilander et al., 1998). Therefore, early detection of AD is crucial for timely intervention, especially for people at higher risk for developing dementia.

Advanced age and well-known susceptibility genes, such as the *APOE* gene encoding apolipoprotein E (ApoE), remain the most significant non-modifiable risk factors for AD (Al-Hamdan et al., 2010; Walker et al., 2017; Jarrar et al., 2023). Based on numerous epidemiological studies, several modifiable vascular and metabolic factors have been linked to increasing the risk of cognitive impairment and AD, such as midlife hypertension, stroke, midlife diabetes, hyperlipidemia, obesity, and depression, in addition to other lifestyle risk factors including smoking, sleep disturbances, and low levels of education (Al-Shammari and Al-Subaie, 1999; Saeed et al., 2011; Bennett and Thomas, 2014; Levin and Vasenina, 2019; Andreescu and Lee, 2020; Kuring et al., 2020; Tinnirello et al., 2021; Zhao et al., 2023). With the continually growing knowledge about the potential role of modifiable risk factors, many researchers focus on modulating the well-known risk factors of AD and exploring new alternative therapeutic approaches. Thus, considering these preventable risk factors is crucial to ensure a healthy lifestyle for older people and help early AD detection.

Recent advanced diagnostic tools have been made targeting AD pathological hallmarks using brain imaging and blood biomarkers. However, those tools display relative limitations, and some current modalities remain limited to the bench. For instance, some cognitive tests are subjective, and interobserver variabilities are unavoidable (Cao et al., 2019). Thus, clinicians find early detection and differentiation between mild cognitive impairment (MCI) and AD challenging. As increasing clinical demand is required for AD diagnosis, artificial intelligence (AI) offers aid methods for predicting and diagnosing dementia (Reid et al., 2006; Cooke and Ancoli-Israel, 2011).

Several studies have used data from magnetic resonance imaging (MRI) of the brain to predict or diagnose different neurodegenerative diseases (Wang et al., 2018; Leong and Abdullah, 2019; Ma et al., 2020; Goenka and Tiwari, 2021; Alqurashi et al., 2022). Moreover, machine learning methods and graph analysis tools have been used to investigate brain functionalities using clinical biomarkers, for which these innovative approaches aid the early diagnosis of dementia (Jeune et al., 2018; Bayahya et al., 2022). On the other hand, only a few studies were focused on AD risk factors alone to be used as predictors for dementia (Bratić et al., 2018; Bin-Hezam and Ward, 2019). Although the current advanced research focuses on the application of machine learning in dementia diagnosis and risk prediction, some models often require extensive data that is not routinely available, such as advanced brain imaging and invasive techniques for biomarkers. Consequently, applying such models is complex and unapplicable for early diagnosis of dementia. Thus, this study aims to develop an AI-based predictive model for early detection of neurocognitive disorders using simple clinical and demographical data extracted from patients’ physical interviews and cognitive testing and not relying on data from imaging studies with minimal cost.

Globally, the number of older people aged 60 years or over has been predicted to double by 2050 (World Health Organization, 2023). As a result, the risk of developing dementia is also expected to increase dramatically (Walker et al., 2017). In the Kingdom of Saudi Arabia (KSA), the aging population is growing, with people aged more than 60 years, around 1.3 million in 2016. The number is expected to rise and reach more than 10 million by 2050 (Jarrar et al., 2023), substantially increasing financial and emotional burdens on families and caregivers. Additionally, the lack of early detection and accurate diagnosis of neurocognitive disorders in adults is considered an issue that needs to be addressed. Therefore, this study aims to analyze and early detection of cognitive decline in older adults in KSA using modern data science approaches, which have rarely been studied. Initially, various factors associated with cognitive decline and dementia were identified. Based on these factors, several models were developed to predict and detect neurocognitive disorders.

## 2 Methods

### 2.1 Survey design and study area

This research conducted a cross-sectional study using in-person interviews with participants recruited from COVID vaccine centers in the Eastern Province of Saudi Arabia. The inclusion criteria were individuals 60 years or older. Participants were excluded from the study if they reported recent head trauma in the past 3 months or were bedbound secondary to any neurological disease. A systematic sampling technique was used to enroll participants by five research assistants. At the start of the study, it was made explicitly clear to patients that their responses would stay anonymous. All participants gave written informed consent, and the study was approved by the Institutional Review Board of Imam Abdulrahman bin Faisal University (IAU) (IRB Number − 2021-01-129).

The data was collected and stored electronically using a secure online platform (Question Pro), which was subsequently de-identified and analyzed. The utilized survey was based on a previously validated survey and enhanced with items selected after thoroughly reviewing the relevant literature (Honda et al., 2021). The questionnaire fundamentally consisted of four sections: the first part consisted of three socio-demographic questions; the second part included four questions measuring the patient’s sleeping routine; the third part consisted of nine questions to explore the patient’s medical history. The Saint Louis University mental status (SLUMS) questionnaire was used in the fourth part of the study to identify the level of the patient’s cognitive problems (Hou et al., 2019). This screening form is composed of 11 short questions scored on a 30-point scale, and the questionnaire covers a wide range of cognitive functions, including memory, attention, orientation, and overall executive function. Based on the SLUMS Examination scores out of 30, the level of the patient’s cognitive problems was categorized as (i) Normal (score 25–30), (ii) MCI (Mild Cognitive Impairment) (score 20–24), (iii) dementia (score 0–19).

The random survey was carried out from May 2020 to January 2021, and data were collected from 343 patients, which is an acceptable sample size for a statistical evaluation to characterize the parameters for observational studies with large population size (Zakir Hossain et al., 2016; Düştegör et al., 2018; Sultana, 2019; Sultana, 2022). There were dropouts in the study.

### 2.2 Statistical analysis

For data analysis, this study used SPSS version 26 and MATLAB version R2022b. The factors pertaining to respondents’ sociodemographic profiles, sleeping habits, medical histories, and the severity of the cognitive issue were examined using descriptive statistics. For categorical variables, the analyses were given as counts and percentages; for quantitative variables, they were presented as means, standard deviations, minimum and maximum values, and interquartile ranges (IQR).

The association between the level of cognitive difficulties and the descriptive categorical features were analyzed using the Chi-square test. A chi-squared test is a statistical hypothesis test where the observed counts in a two-way sample data table are compared with expected counts. The chi-square statistic, which is defined as $\chi^{2}=\sum \frac{{\left(\mathrm{Observed}-\mathrm{Expected}\right)}^{2}}{\mathrm{Expected}}$, is the test statistic used for this comparisom. Here, “observed” and “expected” refer to the observed and expected cell counts for a given cell. The overall r×c cells in the two-way table, where r and c stand for the table’s row and column, respectively, make up the sum. The value of p is the region below the density curve of the ${\chi^{2}}_{\left(r-1\right)\left(c-1\right)}$ distribution to the right of the test statistic. The statistically significant level was set as a value of *p* <0.05.

### 2.3 Description of the proposed machine learning algorithms

Multiple models were created for predicting the cognitive function of the senior population utilizing well-known statistical and machine learning methods, including logistic regression, classification trees ensemble, nearest-neighbor classification, and Bayesian networks. Several experiments were run, and the efficiency and accuracy of the developed models were compared to determine the best predictive models. Finally, based on the model performance, two widely used classifiers (*viz.* logistic regression and SVM) were used to assess patients’ cognitive function. This study also investigated the gender disparities in patients’ cognitive function and created distinct predictive models for men and women. A concise methodological description of these approaches is provided in the following subsections.

#### 2.3.1 Logistic regression model

For the purpose of estimating the extent of dementia, the logistic regression model was created. In the logistic regression method, a categorical response variable is determined by one or more descriptive features. When the categorical response variable only has two possible outcomes (ex. success/failure), binary logistic regression is implemented. In a binomial logistic regression model, the log of odds is used as a dependent variable and is denoted as follows:


$$\mathrm{Odds}=\frac{\mathrm{probability}\;\mathrm{of}\;\mathrm{success}}{\mathrm{probability}\;\mathrm{of}\;\mathrm{failure}}$$

The logit function $f\left(T\right)=\frac{1}{1+e^{-T}}$, where −∞≤T≤∞, can be used to fit data to a model to determine the likelihood of a particular outcome. As T changes from −∞ to ∞, this function, $f\left(T\right)\text{,}$ increases monotonically from 0 to 1. Maximum likelihood estimation is used in the binomial logistic regression algorithm. The first step in this iterative approach is determining the optimal coefficient/weight for each descriptive feature. Then, until there is no more improvement in predicting the response variable for each case, these weights are adjusted repeatedly. Similar to binomial logistic regression, multinomial logistic regression is used to predict multiple outcomes (Jeune et al., 2018). The multinomial logistic regression approach was utilized in this study to determine the degree of cognitive function.

#### 2.3.2 Support vector machines

The SVM is an extensively used supervised machine learning technique, first established by Vapnik et al. using the statistical learning theory (Vapnik, 1995; Adewumi et al., 2016; Li et al., 2017; Alade et al., 2018; Roy and Chakraborty, 2024). The algorithm intends to get the best possible separating hyperplane, which maximizes the most prominent space to the nearby data point of any class, known as the margin. The SVM procedure can be trained for both linear and nonlinear problems. The linear SVM algorithm is used when the data points can be linearly separated. The nonlinear SVM algorithm, on the other hand, is suitable for handling complex and non-separable datasets. The primary goal of this technique is to perform specific nonlinear mapping on the input vectors to transform them into a higher dimensional feature space and then to construct the most prominent classifying hyperplane within that feature space (Cortes and Vapnik, 1995; Brereton and Lloyd, 2010). However, the mapping process usually requires substantial computations and is thus rendered worthless (Mohammadi et al., 2015). In order to resolve this issue, the SVM algorithm applies the kernel trick by using some kernel functions, which helps to project data into a higher dimensional space where the points can be separated linearly.

The kernel function plays a vital role in the SVM model performance. The commonly used kernel functions are linear, polynomial, and Gaussian. The performance of an SVM model varies significantly on the proper selection of the hyperparameters, comprising kernel function, kernel scale, and box constraints. A large kernel scale’s value lends to overfitting, while a small value lends to poor prediction accuracy. The regularization parameter box constraint controls the maximal cost inflicted on margin-violating data points and thereby helps to prevent overfitting. The SVM classifier assigns fewer support vectors as the box constraint increases. However, it may contribute to a more extended training phase.

### 2.4 Performance evaluation of the developed model

The confusion matrix and ROC curve were examined to assess the developed models’ performance. A specific tabular arrangement of the prediction accuracy of a classifier exemplifies the confusion matrix. Typically, each table row demonstrates an observed class, and each column is a predicted class. Several statistical methods of performance evaluation were used for assessing the established models, including prediction accuracy, recall (true positive rate), precision (positive predictive value), and F1 score, as described in Equation (1–4) (Wang et al., 2022).


(1) $$\mathrm{Accuracy}=\frac{\sum_{i}TP_{i}\;}{\mathrm{total}}$$

(2) $$\mathrm{Recall}\;\left(i\right)=\frac{TP_{i}}{TP_{i}+FN_{i}}$$

(3) $$\mathrm{Precision}\;\left(i\right)=\frac{TP_{i}}{\left(TP_{i}+FP_{i}\right)}$$

(4) $$F1\;\mathrm{Score}\;\left(i\right)=\frac{2*\mathrm{Recall}\;\left(i\right)*\mathrm{Precision}\left(i\right)}{\mathrm{Recall}\;\left(i\right)+\mathrm{Precision}\left(i\right)}$$

Here i represents the classes, $P_{i}$ represents the positive/yes class i, $N_{i}$ represents the negative/not class i, T (true) indicates correct classification, and F (false) indicates the wrong classification. The macro-averaged and weighted average of each class’s recall, precision, and F1-score was also calculated to analyze the model’s overall performance.

Additionally, the ROC curve, created by graphing the false positive rate against the true positive rate of a specific class label, explains the investigative potential of a classifier. The area beneath the ROC curve determines how well the classifier performs; the greater the area, the better the accuracy. An outstanding predictive model has an area below the ROC curve that ranges between 0.9 and 1, while a reasonably good predictive model would need this area to range between 0.8 and 0.9 (Aljawad et al., 2017).

## 3 Results

### 3.1 Descriptive statistics

A descriptive analysis of the Socio-demographic variables was conducted (see Table 1). The range of the participant’s ages is from 60 to 90 years, with an average age of 66 years and a standard deviation of 6 years. Sixty-eight percent of the patients are male. According to the respondents’ educational backgrounds, only roughly 8% have postgraduate degrees, 29% have undergraduate degrees, 22% have completed high school, and 41% have not. This shows that the majority of participants had low educational backgrounds. The patient’s medical condition was also analyzed (see Table 1). The results show that 48% of patients have diabetes, 56% patient have hypertension, 18% have coronary artery diseases, 8% have a stroke, less than 3% have depression, 7% have anxiety, 44% have chronic pains, 28% take medications for chronic pain, and about 7% take sleeping pills.

**Table 1:** Descriptive analysis of the feature variables and the chi-square test result of the categorical features with the cognitive function.

Numerical variables	Mean	Median	SD	
Age	66	65	6	
Bedtime	10:13 PM	10:00 PM	–	
Wakeup time	7:00 AM	7:30 AM	–	
Sleep at night (hour)	6	6	1.8	
Nap (minutes)	44	30	52	
Categorical variables	Categories	Count	%	Chi-square (*p*-value)
Sex	Male	233	67.9	26.651 (0.000^*^)
	Female	110	32.1	
Education	Not high school	142	41.4	84.714 (0.000*)
	High school	74	21.6	
	Undergraduate	100	29.2	
	Postgraduate	27	7.9	
Diabetes	No	179	52.2	11.240 (0.004^*^)
	Yes	164	47.8	
Hypertension	No	152	44.3	1.904 (0.386)
	Yes	191	55.7	
Coronary artery diseases	No	280	81.6	6.813 (0.033^*^)
	Yes	63	18.4	
Stroke	No	317	92.4	8.091 (0.018^*^)
	Yes	26	7.6	
Depression	No	335	97.7	1.216 (0.544)
	Yes	8	2.3	
Anxiety	No	318	92.7	1.463 (0.481)
	Yes	25	7.3	
Chronic pain	No	192	56	3.403 (0.182)
	Yes	151	44	
Take pills for chronic pain	No	248	72.3	1.393 (0.498)
	Yes	95	27.7	
Take sleep pills	No	320	93.3	1.705 (0.426)
	Yes	23	6.7	

*Significant at the 0.05 level.

**Figure 1:**
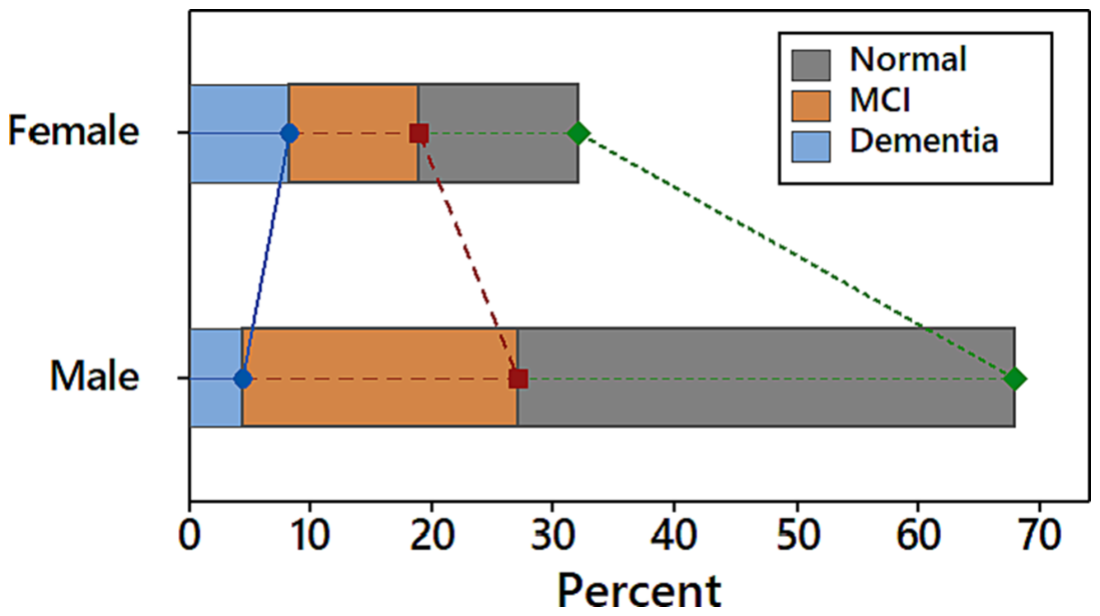
Distribution of cognitive function levels among participants stratified by gender.

The Saint Louis University mental status (SLUMS) questionnaire was used to determine the severity of the patient’s cognitive decline. Based on the result of SLUMS, 54% of patients have normal cognitive function, 34% have MCI, and 12% have dementia. Males are more likely than females to have both normal cognitive function and MCI, while females are more likely to have dementia (see Figure 1).

According to the chi-square test results, the significant factors of cognitive function are sex, education, diabetes, coronary artery diseases, and stroke (all *p* < 0.05). Individuals with lower educational levels have a greater risk of dementia than individuals with higher academic levels. Diabetes patients had a greater rate of cognitive decline (40.2% MCI and 15.2% dementia) than non-diabetic patients (27.4% MCI and 10.1% dementia). Similar findings have been obtained for other diseases like hypertension, coronary artery disease, stroke, depression, anxiety, and chronic pain. Among the patients taking pills for chronic pain, about 16% have dementia, while 11% have dementia among patients who are not taking medications for chronic pain. Similarly, individuals who take sleeping pills are more likely to have dementia (17%) than those who do not (12.2%).

### 3.2 Developed models

The data were randomly allocated to training (80%) and testing (20%) datasets. The model was built using the training data, and the testing data was used to measure the prediction errors and avoid overtraining. The accuracy of the developed models determined by the training and testing datasets did not show any notable differences, implying that all of the developed models are neither overfitted nor underfitted.

#### 3.2.1 Logistic regression model

The model-relevant information of the developed logistic regression model was analyzed. The value of p of the Likelihood Ratio Tests is very small ($\chi^{2}=236.559,p<0.000)\text{,}$ indicating a significant difference between the “intercept only” and developed model. The pseudo-R-square measures were analyzed to estimate the variation in the dependent variable that the model can explain. The results indicate that the proposed logistic regression model explained between 49.8% (Cox and Snell R square) and 58.4% (Nagelkerke R square) of the variance in the cognitive level.

#### 3.2.2 Support vector machine model

The Bayesian hyperparameter optimization algorithm was employed to determine the kernel function, optimal value of box constraint, and kernel parameter scale. Further, several experiments were conducted to construct the best SVM models. The maximum accuracy for the model of all patients was achieved using the Gaussian kernel function with the box constraint and Kernel parameter scale of 37 and 0.1, respectively (see Table 2). The highest accuracy for the models of males and females was also achieved using the Gaussian kernel function with a kernel scale of 0.1. The optimized value of the box constraints for males and females are 41 and 23.5, respectively (see Table 2).

**Table 2:** Optimized hyperparameters of the developed SVM models.

	Parameter	Value
SVM Model for all	Kernel type	Gaussian
	Kernel scale	0.1
	Box constraints	37
SVM Model for male	Kernel type	Gaussian
	Kernel scale	0.1
	Box constraints	41
SVM Model for female	Kernel type	Gaussian
	Kernel scale	0.1
	Box constraints	23.5

### 3.3 Performance evaluation and model comparison

The confusion matrix and ROC curve were analyzed to evaluate the model. Based on the confusion matrix, the developed logistic regression model for all patients has an accuracy of about 71.43%, while the developed logistic regression model for males and females has an accuracy of 73.8 and 100%, respectively (shown in Table 3). However, the developed SVM model for all patients has an accuracy of 93.3%, while the SVM models for males and females have an accuracy of 94.4 and 95.5%, respectively.

**Table 3:** Confusion matrix for the developed logistic regression and SVM models.

			All			Male			Female		
			Predicted			Predicted			Predicted		
			Normal	MCI	Dementia	Normal	MCI	Dementia	Normal	MCI	Dementia
Logistic regression	Observed	Normal	153	29	3	121	18	1	45	0	0
		MCI	43	65	7	33	43	2	0	37	0
		Dementia	3	13	27	4	3	8	0	0	28
			Accuracy: 71.4%			Accuracy: 73.8%			Accuracy: 100.0%		
SVM	Observed	Normal	173	11	1	137	3	0	43	2	0
		MCI	5	110	0	9	69	0	1	34	2
		Dementia	1	5	37	0	1	14	0	0	28
			Accuracy: 93.3%			Accuracy: 94.4%			Accuracy: 95.5%		

Precision is the percentage of correctly identifying a cognitive level, while recall is the percentage of identifying all instances of a particular cognitive level. The F1-score is their harmonic mean, while support designates the number of occurrences of each level. In general, the desirable classifier performance is to exhibit the same levels of precision and recall for each class, with both metrics being as high as possible. The macro-average and weighted-averages of precision, recall, and F1-score were also computed. Based on the results shown in Table 4, it is evident that the developed logistic regression model achieves a satisfactory performance, with the minor exception of the recall metric on MCI class. Precision, recall, and F1-scores based on the logistic regression model for males are acceptable, with the minor exception of the recall metric on MCI class. While all these scores for females are 100%, indicating an outstanding performance for this developed model. However, the values of these performance indicators for all the developed SVM models are greater than 90%, except the precision of MCI class for the SVM model based on all patients is 87.3% (Table 4).

**Table 4:** Performance evaluation of the developed logistic regression and SVM models.

		All			Male			Female		
		Precision	Recall	F1-score	Precision	Recall	F1-score	Precision	Recall	F1-score
Logistic Regression	Normal	76.9	82.7	79.7	76.6	86.4	81.2	100	100	100
	MCI	60.7	56.5	58.6	67.2	55.1	60.6	100	100	100
	Dementia	73.0	62.8	67.5	72.7	53.3	61.5	100	100	100
	Macro-averaged	70.2	67.3	68.6	72.2	65.0	67.8	100	100	100
	Weighted-averaged	71.0	71.4	71.1	73.2	73.8	73.0	100	100	100
SVM	Normal	96.6	93.5	95.1	93.8	97.9	95.8	97.7	95.6	96.6
	MCI	87.3	95.7	91.3	94.5	88.5	91.4	94.4	91.9	93.2
	Dementia	97.4	86.0	91.4	100.0	93.3	96.6	93.3	100.0	96.6
	Macro-averaged	93.8	91.7	92.6	96.1	93.2	94.6	95.2	95.8	95.4
	Weighted-averaged	93.6	93.3	93.3	94.5	94.4	94.4	95.5	95.5	95.4

**Figure 2:**
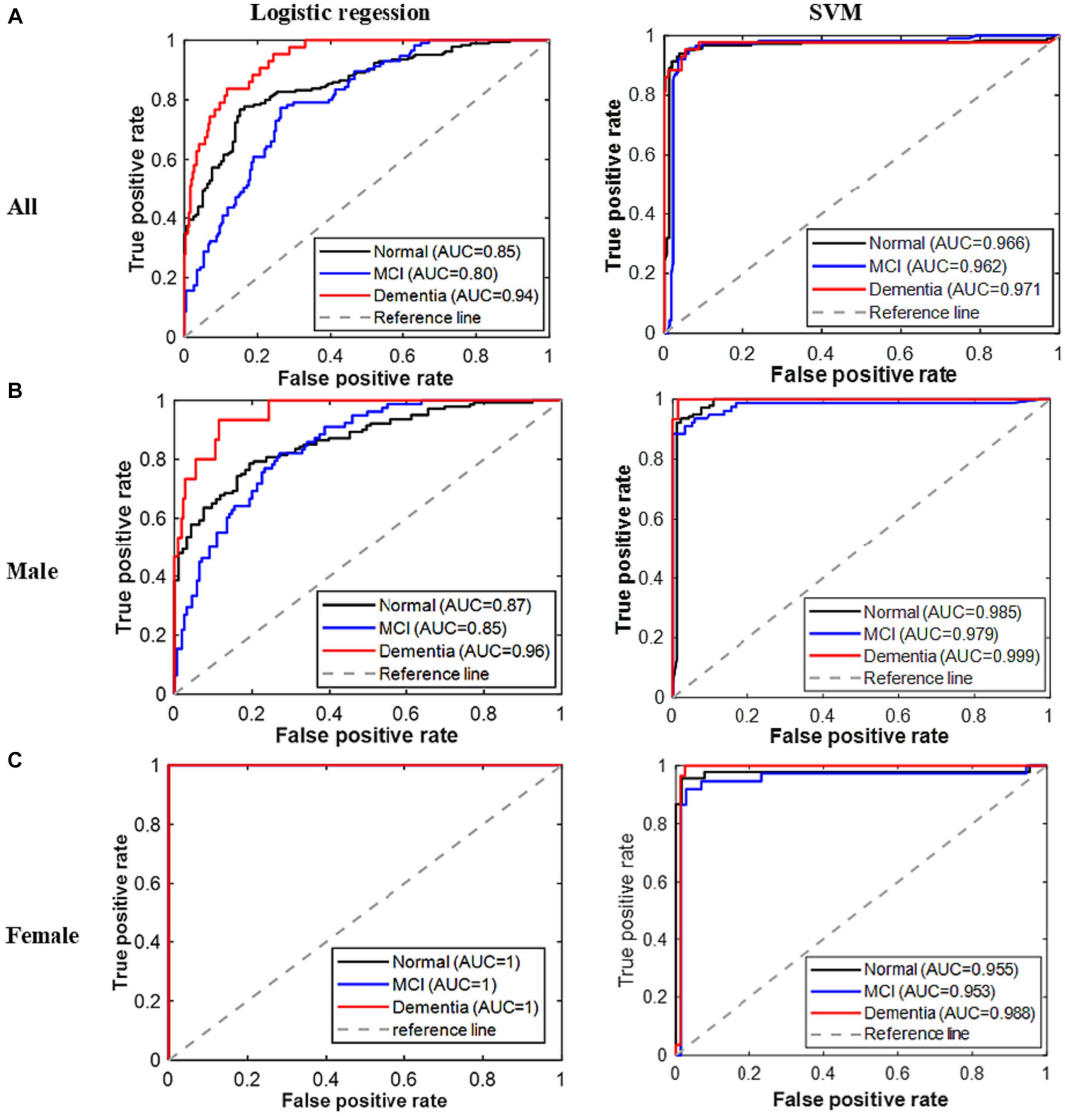
ROC curves for all classes of cognitive problems based on the developed models. **(A)** All. **(B)** Male. **(C)** Female.

Figure 2 displays the ROC curves for all three classes of the established models. As expected, the area underneath the ROC curves for all classes in the logistic regression model for all patients is greater than 0.8. However, the AUC for the dementia class is higher (>0.94) in this model. The areas underneath the ROC curves of all classes are greater than 0.85 for the males’ logistic regression model, while these are exactly 1 for the females’ logistic regression model. On the other hand, the areas underneath the ROC curves for all the developed SVM models are greater than 0.95. Therefore, based on the result of all these performance indicators, the performances of the developed models are remarkable; however, the reported SVM model performed the best.

**Figure 3:**
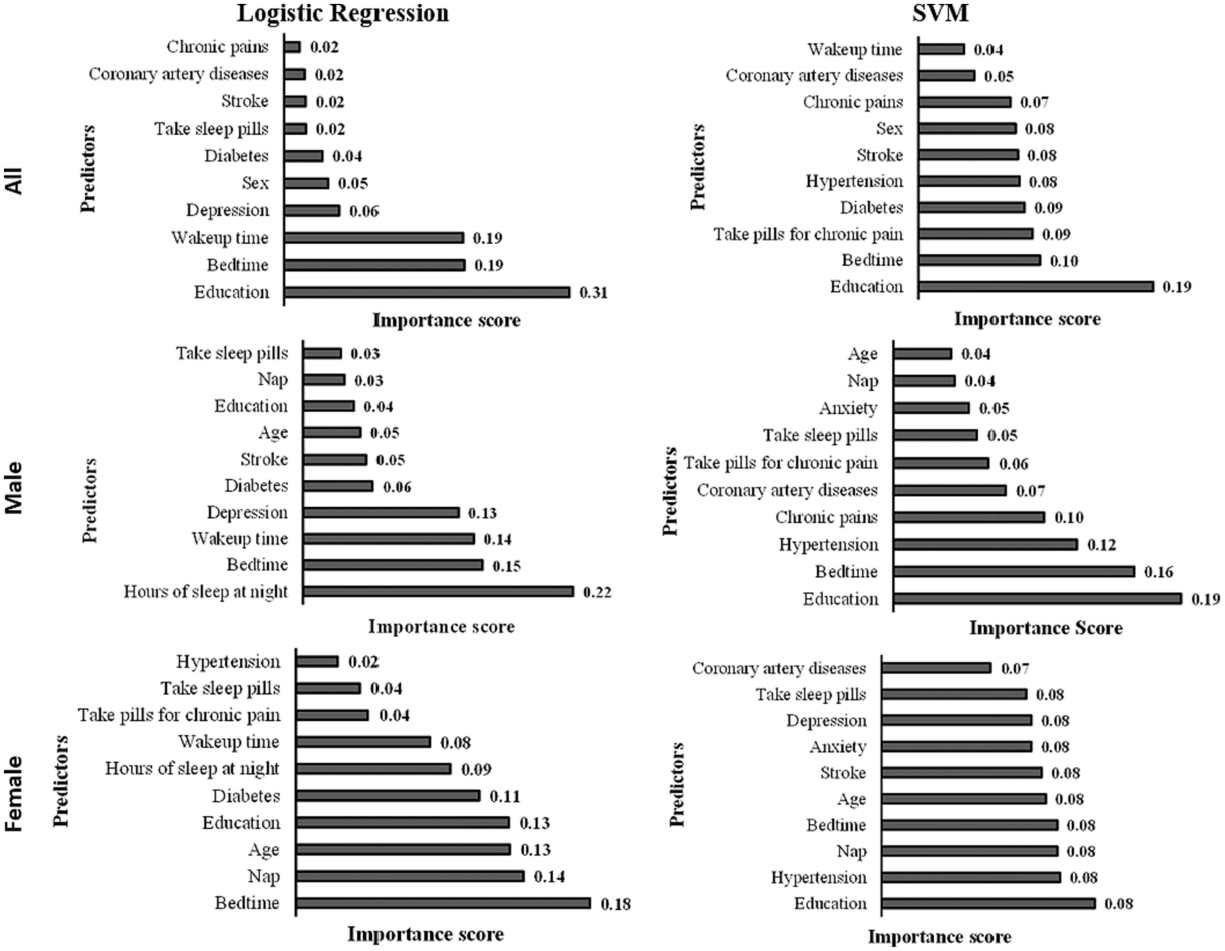
Predictor importance chart in the developed models.

### 3.4 Relative importance of the predictor

The predictor importance chart was created to determine the effect of each predictor on the level of cognitive functions based on the developed logistic regression model (see Figure 3). The top 10 essential predictors based on their effect on cognitive functions in the logistic regression model are education, bedtime, wake-up time, depression, sex, diabetes, taking sleep pills, stroke, coronary artery diseases, and chronic pains. While the top 10 important predictors based on their effect on the cognitive function in the logistic regression model for males are hours of sleep at night, bedtime, wake-up time, depression, diabetes, stroke, age, education, nap, and taking sleep pills; and the top 10 significant predictors of the developed model for females are bedtime, nap, age, education, diabetes, hours of sleep at night, wake-up time, taking pills for chronic pain, taking sleep pills, and hypertension.

Based on the predictor important chart (shown in Figure 3), the top 10 significant predictors based on the developed SVM model are education, bedtime, taking pills for chronic pain, diabetes, stroke, sex, chronic pains, coronary artery diseases, and wake-up time. For the SVM males’ model, the top 10 important predictors are education, bedtime, hypertension, chronic pains, coronary artery diseases, taking pills for chronic pain, taking sleep pills, anxiety, nap, and age. In comparison, the top 10 significant predictors of the developed SVM females’ model are education, hypertension, nap, bedtime, age, stroke, anxiety, depression, taking sleep pills, and coronary artery diseases.

## 4 Discussion

This study highlighted the utilization of AI in predicting cognitive decline among healthy participants from the Eastern Province of Saudi Arabia, which has rarely been studied. The artificial intelligence-based algorithm SVM model was developed to predict cognitive decline and dementia in Saudi patients and compared the performance with the widely used probability-based ML approach (logistic regression). Several performance measures were applied to evaluate the developed models’ performances, including prediction accuracy, precision, recall, F1-score, and AUC. Both proposed models had high prediction accuracy (>71%) and a high value of AUC (>0.80) for all three classes, suggesting excellent prediction performance. The SVM model, in contrast, produced the best prediction performance (Accuracy >93% and AUC > 0.96 for all three classes), which is the highest accuracy for a three-class model based on literature. Separate predictive models for males and females were also developed using logistic regression and SVM algorithms, and the model performances were compared. Both models show excellent prediction performance for males and females; however, SVM performed outstandingly (accuracy of 94.4% for males and 95.5% for females). This study achieved these satisfactory results based on the optimal use of the feature selection methods, hyperparameter optimization, and the machine learning algorithm SVM that uses the kernel trick technique and achieves the highest prediction performance.

It has been reported that higher cognitive functions are positively correlated with the number of years of formal education as it has been proposed that early childhood learned cognitive skills may persist into older age (Lövdén et al., 2020). Also, a recent study by Rosselli et al. showed that a higher level of education is a protective factor against future cognitive decline (Rosselli et al., 2022). The results of these studies are consistent with what we have concluded. However, this result contrasts with some other studies (García-Magariño et al., 2020; Palacios-Navarro et al., 2022), and few reviews state that there was no clear evidence of a significant relationship between the participants’ level of education and performance on memory. Thus, future research that entirely focuses on determining the effects of education on neurocognitive disorders can be conducted.

The presence of comorbidity, precisely chronic diseases, has been known to play an important role in the decline of cognitive functions. For example, diabetes mellitus and hypertension are highly associated with the presence of vascular dementia and Alzheimer disease in the elderly (Biessels and Despa, 2018). Recently, it has been proposed that high blood pressure may alter both the anatomy and physiology of the brain, specifically leading to cerebral vessel remodeling where the blood vessels of the brain are unable to clear potentially harmful proteins such as β-amyloid (Walker et al., 2017). Many polysomnography studies indicated that sleep cycle is also an important factor in the elderly with a reduction in slow-wave sleep and more fragmented and lighter sleep leading to excessive daytime sleepiness (Cooke and Ancoli-Israel, 2011). Another common sleep disturbance in the elderly is insomnia, defined as the inability to initiate or maintain sleep (Reid et al., 2006). Alqurashi et al. reported that in a sample of the elderly population in Saudi Arabia, cognitive decline was apparent among extended nappers (who nap >90 min per day) (Alqurashi et al., 2022). Also, a recent study showed an inverted U-shaped association between sleep duration and cognitive decline in the elderly, where cognitive decline was apparent in those with insufficient (≤4 h per night) or excessive (≥10 h per night) sleep duration (Ma et al., 2020). The current study reported that cognitive decline was associated with the elderly population who are on sleeping pills, suggesting that although sleep disturbances are common in older adults, this group should be monitored carefully for any early signs or symptoms of cognitive decline.

Several studies investigated dementia risk using the classical Cox regression model (Tang et al., 2015; Albrecht et al., 2018; Jammeh et al., 2018; Hou et al., 2019; Licher et al., 2019; Bock et al., 2020; Fukunishi et al., 2020; Stephan et al., 2020). The Cox regression algorithm was used to develop a 3-year dementia risk score in individuals aged 55 years and older with mild cognitive impairment (MCI) in Canada (Hou et al., 2019). This efficient and clinically useful score is suitable to apply in a care setting to predict dementia risk in individuals with MCI without having advanced imaging, cerebrospinal fluid analysis, or neuropsychological testing. Using structured expert elicitation (SEE) methodology, the experts’ opinions of significant features of 3-year dementia risk in individuals with MCI were evaluated (Tang et al., 2015). The Bayesian Cox regression method was used to combine patient data and expert knowledge for determining dementia risk scores in patients with MCI. The Cox proportional-hazards regression analysis was used to create the multivariable prediction model using predictors obtained from primary care settings to develop a model for the prediction of dementia risk in Japan (Stephan et al., 2020). The developed model was converted into a condensed scoring scheme based on the beta coefficient. The Harrell’s C-statistic and calibration plots were used in this study to assess the developed model’s discrimination and calibration, respectively. The authors claimed that The proposed risk prediction model is practical and valuable for primary-care settings to identify people at high risk for future dementia because the constructed model and simplified score exhibited effective discrimination and calibration.

A limited number of studies used other modern data science algorithms (*viz.* classification trees ensemble, nearest-neighbor classification, Bayesian network, artificial neural network, and support vector machine) to predict the risk of dementia (Barnes and Yaffe, 2009; Tang et al., 2015; Walters et al., 2016; Jeune et al., 2018; Nori et al., 2019; Gill et al., 2020; Kumar et al., 2021; Reinke et al., 2022). Logistic regression (LR), gradient boosting (GBM), and random forests (RFs) were used to develop predictive models to investigate whether the German claims data are suitable for dementia risk prediction (Gill et al., 2020). Twenty-three age-related diseases, 212 medical prescriptions, and 87 surgery codes, as well as age and sex, were used as potential features. The results demonstrated that discriminatory power was moderate for the developed LR and GBM and lowered for RF. The GBM had the best model calibration. This study identified antipsychotic medications, cerebrovascular disease, and a less-established specific antibacterial medical prescription as important predictors. The developed models from German claims data have acceptable accuracy and may provide cost-effective decision support for early dementia screening.

Further, few researchers used imaging datasets (Wang et al., 2018; Leong and Abdullah, 2019; Ma et al., 2020; Goenka and Tiwari, 2021; Alqurashi et al., 2022), which added to the difficulty of gathering data and the inconvenience of non-technical people to utilize highly complex assembled models. In addition, it was observed that most of the studies attained low sensitivity rates, while other studies reached inadequate classification accuracy. In order to overcome these limitations, simple clinical data extracted from physical interviews was used to develop the proposed AI-based model, and considerably high accuracy and recall rates were achieved with minimal cost and computation time.

A delay in diagnosing neurocognitive disorders leads to rapid disease progression and may threaten the patients’ lives as the disease progresses over time. Using simple clinical data in the proposed models benefits the early diagnosis of neurocognitive disorders due to its fewer risks and expenses than MRI scans. For example, people who have medical equipment implanted, such as pacemakers, are not allowed to have MRI scans because of the risk of burns, unwanted movements, and malfunctions. In addition to other safety issues, MRI scans could harm the patient’s body if performed improperly. Consequently, hospitals can benefit from the preventative prediction of cognitive decline at a low cost by developing an accurate AI-based model utilizing clinical data.

## 5 Conclusion

Dementia has now been recognized as one of the Global Challenges. It will substantially increase financial and emotional burdens on families and caregivers; thus, early diagnosis of cognitive decline is crucial for timely intervention, especially for people at higher risk for developing dementia. Thus this study commenced to develop artificial intelligence-based models that preventively predict dementia to enhance the pre-emption measures and reduce the mortality rate induced by this disease. Two algorithms, *viz.*, support vector machine (SVM) and logistic regression (LR), were trained using a Saudi dataset collected from COVID-19 vaccine centers in the Eastern Province. Additionally, distinct prediction models for males and females were created after analyzing the gender-based cognitive function of the patients. All the developed models show good prediction capability with accuracy greater than 71%. However, the generated SVM models performed the best, with an accuracy of 93.3% for all patients, 94.4% for men exclusively, and 95.5% for women-only models. The top 10 significant predictors based on the developed SVM model for all patients are ranked as (Winblad et al., 2016) education, (Cummings-Vaughn et al., 2014) bedtime, (Ferencz and Gerritsen, 2015) taking pills for chronic pain, (Wang et al., 2022) diabetes, (Wang et al., 2021) stroke, (Doblhammer et al., 2022) gender, (Goerdten et al., 2019) chronic pains, (Kilander et al., 1998) coronary artery diseases, and (Walker et al., 2017) wake-up time. The key novelty of this study is the early detection of cognitive function without relying on the radiological findings. Subsequently, future work may incorporate investigating approaches to reduce the number of features while maintaining high accuracy. The suggested methods can also be expanded to address other chronic syndromes. Further, while developing AI-based models addressing medical problems, using the least computational techniques with datasets available is recommended to facilitate adapting the preemptive prediction tool in most healthcare facilities while addressing the economic concerns.

## Data availability statement

The raw data supporting the conclusions of this article will be made available by the authors, without undue reservation.

## Ethics statement

The studies involving humans were approved by the Institutional Review Board of Imam Abdulrahman bin Faisal University (IAU) (IRB Number − 2021-01-129). The studies were conducted in accordance with the local legislation and institutional requirements. The participants provided their written informed consent to participate in this study.

## Author contributions

KA: Writing – original draft, Writing – review & editing, Conceptualization, Data curation, Resources. NS: Conceptualization, Formal analysis, Investigation, Methodology, Software, Visualization, Writing – original draft, Writing – review & editing. NAM: Conceptualization, Writing – original draft, Writing – review & editing. AA: Conceptualization, Writing – original draft, Writing – review & editing. NAS: Conceptualization, Writing – original draft, Writing – review & editing. MB: Formal analysis, Software, Visualization, Writing – original draft. KA: Formal analysis, Software, Visualization, Writing – original draft. AB: Formal analysis, Software, Visualization, Writing – original draft. ZA: Formal analysis, Software, Visualization, Writing – original draft. DB: Formal analysis, Software, Visualization, Writing – original draft. SSA: Writing – original draft, Writing – review & editing. SA: Writing – original draft, Writing – review & editing. YA: Writing – original draft, Writing – review & editing. KG: Writing – original draft, Writing – review & editing.
